# A deep learning framework for automated fracture detection and localization in radiographic images using convolutional neural networks

**DOI:** 10.3389/fmed.2026.1806133

**Published:** 2026-04-13

**Authors:** Jiangdong Lu, Jie Ding, Penglong Wang, Boyan Mi, Panyu Zhou, Feng Zheng

**Affiliations:** 1 Department of Computer and Simulation Technology, Naval Medical University, Shanghai, China; 2 Department of Emergency Surgery, Changhai Hospital, Naval Medical University, Shanghai, China; 3 Department of Orthopedics, Changhai Hospital, Naval Medical University, Shanghai, China

**Keywords:** accuracy, area under receiver operating characteristic curve, average precision, brier score, convolutional neural networks, detectors, EfficientNet-B0, F1-score

## Abstract

**Background:**

Fracture detecting and localizing in radiographic images is essential to enhance the effectiveness of the diagnosis of trauma and allow the image to be interpreted. Despite the potential potential of the deep learning in musculoskeletal imaging, the quality of classification results and the stability of localization are significant issues.

**Purpose:**

The purpose of the work is to design and test a deep learning system that fractures radiographic images and localizes them with the use of convolutional neural networks and object detection on the basis of YOLOv8.

**Methods:**

A retrospective secondary data analysis was done based on publicly available, de-identified radiographic data. In fracture classification, three transfer-learning backbones were analyzed: ResNet18, MobileNetV3-Small, and EfficientNet-B0, which were trained on repeated stratified cross-validation with early stopping. The evaluation of model performance was with area under receiver operating characteristic curve (AUROC), average precision (AP), Brier score, accuracy, precision, recall, and specificity and F1-score. The temperature scaling was used to perform probability calibration and the nested threshold optimization to compare the performance at various operating points. To localise fractures, Precision, recall, mAP, 0.5, and mAP, 0.5:0.95 were used to compare and train YOLOv8n, YOLOv8s and YOLOv8m detectors on both validation and test sets.

**Results:**

MobileNetV3-Small was the top-performing backbone in terms of overall performance, though the classification discrimination was generally low. Calibration analysis was used to show that probability distribution and reliability properties changed with the scaling of temperature and threshold optimization revealed significant differences in sensitivity, precision, specificity, and F1-score with different decision cutoffs. According to the localization experiment, YOLOv8 showed variability in the performance of the detector variants, with the largest test-set mAPs at 0.5 and the largest variation in classes across anatomical fracture types. These results show that the element of localization in the framework was better and more regular compared to the element of classification in the current experimental setup.

**Conclusion:**

The presented framework offers a combined method of fracture classification, calibration of probability, threshold analysis and radiographic localization. Although the classification aspect demonstrated poor discriminative accuracy, the localization outcomes using the YOLOv8 were relatively better in this scenario, which justifies the usefulness of detector-based fracture localization in this context. Clinical translation will be subject to further external validation, prospective assessment, and comparison of experts and readers.

## Introduction

Bone fractures are one of the most frequent injuries treated in emergencies, trauma, and surgical care, and the correct localization of the fracture areas can be the basis of timely treatment and the best patient outcomes (1). In spite of the fact that the traditional interpretation of radiographs continues to be the clinical standard, the visual assessment is already time-consuming, and it also has inter-observer variation especially in fine or overlapping fracture appearances (2). To overcome those drawbacks, deep learning (DL) techniques have been more recently applied in medical imaging tasks, showing significant advancements in fracture detection accuracy and diagnostic efficiency over the conventional manual methods (3).

Recent advances in convolutional neural networks (CNNs) and object-detecting designs have facilitated the fragmentation of fractures at once during the localization, which elevates the possibility of artificial radiographic interpretation to aid clinical practice (4). Preliminary studies based on object detection networks like YOLOv5 and its derivatives have demonstrated positive results in terms of fracture region localization in X-ray images with high mean average precision values, which confirms the potential of real-time fracture localization (5). More enhanced models, which combine attention and multi-scale feature extraction, have also enhanced detection and localization strengths across a wide range of anatomical locations (6). Although it has progressed to this level, a number of drawbacks on implementing the detection performance with sound confidence estimates, clinically consistent decision thresholds, and broad interpretability continue to pose a major challenge to safe implementation in the patient care setting.

Similar studies have shown that the state-of-the-art performance of the DL models in the classification of fractures can be reached by advanced architecture and the use of ensemble learning (7). But most of these frameworks are only based on classification, or they do not have real-time localization capacity, which limits its translational preparedness. Moreover, the lack of well-calibrated probability output of most DL systems is an obstacle to clinical trust, because uncalibrated scores of confidence may mislead clinicians by creating an illusion of model certainty (8). Other approaches to improve probabilistic reliability like temperature scaling and confidence threshold optimization have come in as viable mechanisms, but their use in conjunction with real-time localization has not been implemented in holistic systems.

Other important factors to clinician acceptance of automated imaging tools include explainability and interpretability. Grad-CAM and saliency map methods can be used to provide a visual explanation of how a model made its decisions, and can show which parts of the image actually affected the predictions, making the results of the algorithms more consistent with clinical reasoning (9). Interpretability serves a twofold purpose, both in revealing common failure configurations, e.g., false positive localizations in complicated anatomical areas, and in building trust in radiologists who need clear explanations as to why automatics are making decisions.

Multi-anatomical datasets with bounding box annotations, which are large, are an excellent basis on which to build and test fracture localization models in a wide range of clinical conditions (8). However, it is common in existing literature to consider calibration, thresholding, and interpretability as independent auxiliary analyses, instead of part of a broader framework. Consequently, there is still a requirement of comprehensive, real-time able systems with built in, precise localization, optimised estimates of confidence, threshold optimization, based on clinical priorities, and explainability.

This paper fills these gaps by suggesting a deep-learning-based automated fracture detection and localization model in radiographic images. We will use a combination of a YOLOv8 object detector to localize in real-time and calibration with optimization of the threshold to maximize probabilistic reliability. We also add interpretability analyses in order to visualize and interpret model behaviour. We focus on enhancing the clinical usability and reliability of DL systems to diagnose fractures and provide surgical advice by locating, calibrating, and detecting through one clinical pipeline.

## Methods

### Design of study and data sources

This study was made as a retrospective secondary analysis of publicly available, de-identified radiographic data sets for automated classification and localization of fractures. Two complementary sets of data were used. For classification of images at the fracture level, the radiographs were separated in class-labelled directories and were used to train convolutional neural network-based models for binary fracture detection. For spatial localization, a separate object detection data set with YOLO-format annotations was used, in which regions of fractures were annotated by normalized bounding boxes and assigned to one of four anatomical classes (elbow, finger, forearm and wrist fracture). The datasets have been used only for a methodological model development and evaluation and no direct patient recruitment or personally identifiable information have been involved.

### Image pre-processing and augmentation

For the classification experiments, all the images were converted to three-channel RGB format and resized to a common input resolution of 224 × 224 pixels. Pixel intensities were normalized with ImageNet mean and standard deviation values to aid transfer learning with pretrained convolutional backbones. During training, on-the-fly augmentation was used, with small angle random rotations and horizontal flipping, in order to develop robustness to variation in positioning and acquisition. For evaluation, only deterministic resizing and normalization were used. For the localization experiments, the input resolution of the radiographs was resized while maintaining annotation compatibility, and augmentation during YOLOv8 training was applied using horizontal flipping, small rotations, translation, scaling, and colour space perturbations to enhance generalization across radiographic appearances.

### Classification model development

The fracture-classification part was made based on transfer learning by using three convolutional neural network architectures ResNet18, MobileNetV3-Small, and EfficientNet-B0. The last classification part of each pretrained network was replaced to fit the amount of classes in the fracture classification task. To increase methodological rigor, compared to the initial pilot experiments, model development was done with stratified 5-fold cross validation using the full available classification dataset, and each model was repeated for 3 random seeds. This gave rise to several fold level evaluations in order to create more stable estimation of predictive performance. Models were optimised by using Adam optimizer with a learning rate of 1×-4 and cross entropy loss. Training was permitted to continue for up to 30 epochs with early stopping according to validation AUROC, using a patience criterion to avoid unnecessary overtraining. The best performing checkpoint from each fold was kept for the final fold level evaluation.

### Classification performance evaluation

For every fold and seed, probabilistic prediction was generated on the held out validation partition. Classification performance was summarized in terms of area under the receiver operating character curve (AUROC), average precision (AP), Brier score, accuracy, precision, recall, specificity and F1-score. Aggregate performance for the different folds and seeds was summarized with mean values and bootstrap-based 95% confidence intervals. In addition, there were paired fold-level differences between architectures that were computed to enable comparative evaluation of competing backbones under matched validation conditions. Learning curves for training loss, validation loss, validation AUROC, and validation AP were also saved for examining the convergence behaviour and to record the impact of early stopping.

### Probability calibration

In an attempt to enhance the reliability of predicted probabilities, *post hoc* temperature scaling was performed on the best performing classification model. Rather than fitting and testing calibration on the same small validation subset, instead, calibration used out-of-fold predictions generated in cross-validation. The out-of-fold probabilities were pooled and divided into calibration subset and independent evaluation subset using stratified sampling. Temperature scaling was fitted on the calibration subset using the minimum of negative log-likelihood, and applied on the independent evaluation subset. Calibration quality before and after scaling using AUROC, average precision, Brier score, expected calibration error (ECE), maximum calibration error (MCE) and calibration-in-the-large (CITL). In order to visualize the effect of calibration on the probabilistic output behaviour, reliability diagrams with bin counts and probability histograms were also generated.

### Threshold optimization

As fracture-screening and confirmatory-use scenarios might be more favourable for different operating points, threshold optimization was done as a separate evaluation step. Confidence thresholds were searched over a predefined grid from 0.10 to 0.90. To avoid optimistic performance estimates, the threshold selection was performed in a nested fashion: for each of the outer held-out runs, the optimal threshold was selected on the remaining development runs based on maximal F1-score, with sensitivity and precision as secondary criteria in the case of ties. The selected threshold was then applied once to the held out run. Final performance threshold-dependent performance was summarized using accuracy, precision, sensitivity, specificity, F1-score, AUROC, and AP, and bootstrap-based confidence intervals and threshold-performance curves.

### Fracture localization using YOLOv8

The fracture-localization part was implemented based on the YOLOv8 framework of Ultralytics. Because the reviewer asked for more comparative analysis, localization was not restricted to only one detector setup, but rather that three detectors in this case were tested, which were one-stage detectors: YOLOv8n, YOLOv8s and YOLOv8m. The localization dataset was split as training, validation, and test data with YOLO format bounding box annotation for elbow, finger, forearm, and wrist fractures. All detectors were trained with supervised optimization for up to 100 epochs with the early stopping learning rate, also, model selection was selected from validation performance. The optimization algorithm used in the training included Adam optimization, cosine learning rate schedule, horizontal flipping, scaling, translation, and mild geometrical and photometric augmentation. This comparison made it possible to benchmark lightweight, intermediate, and higher-capacity versions of YOLOv8 under the same detection task and dataset structure.

### Localization evaluation

Localization performance was evaluated on validation and held-out test sets using common metrics of object detection: precision, recall, mean average precision at IoU = 0.5 (mAP@0.5) and mean average precision for IoU threshold ranging from 0.5 to 0.95 (mAP@0.5:0.95). The extracted per-class performance was extracted where available, and a class-support summary was produced to record the number of annotated fracture instances in each of the categories of anatomy. In addition to the comparison on the detector level of YOLOv8n, YOLOv8s, and YOLOv8m, the qualitative visual assessment of the predicted bounding boxes was done to study the localization behaviour for radiographs with variable anatomy, overlapping structures, and orthopaedic hardware.

### Computational environment and reproducibility

All the analyses have been carried out in Python, using PyTorch, Torchvision, Scikit-learn, Matplotlib, and Ultralytics YOLO version 8 framework. Classification experiments were conducted using GPU acceleration when available, and automatic mixed precision was used for efficiency on compatible hardware support. Random seeds were fixed for repeated runs to increase reproducibility and fold-wise outputs, probability files, calibration summaries, learning curves, and localization results were saved for downstream statistical analysis and figure generation.

### Ethical considerations

This study was granted ethical exemption by the Institutional Review Board of Naval Medical University (Shanghai, China) as it involved only secondary analysis of publicly available, de-identified radiographic datasets. No direct patient contact, recruitment, or personally identifiable information was accessed.

## Results

### Dataset characteristics and pre-processing audit

**Figure 1:**
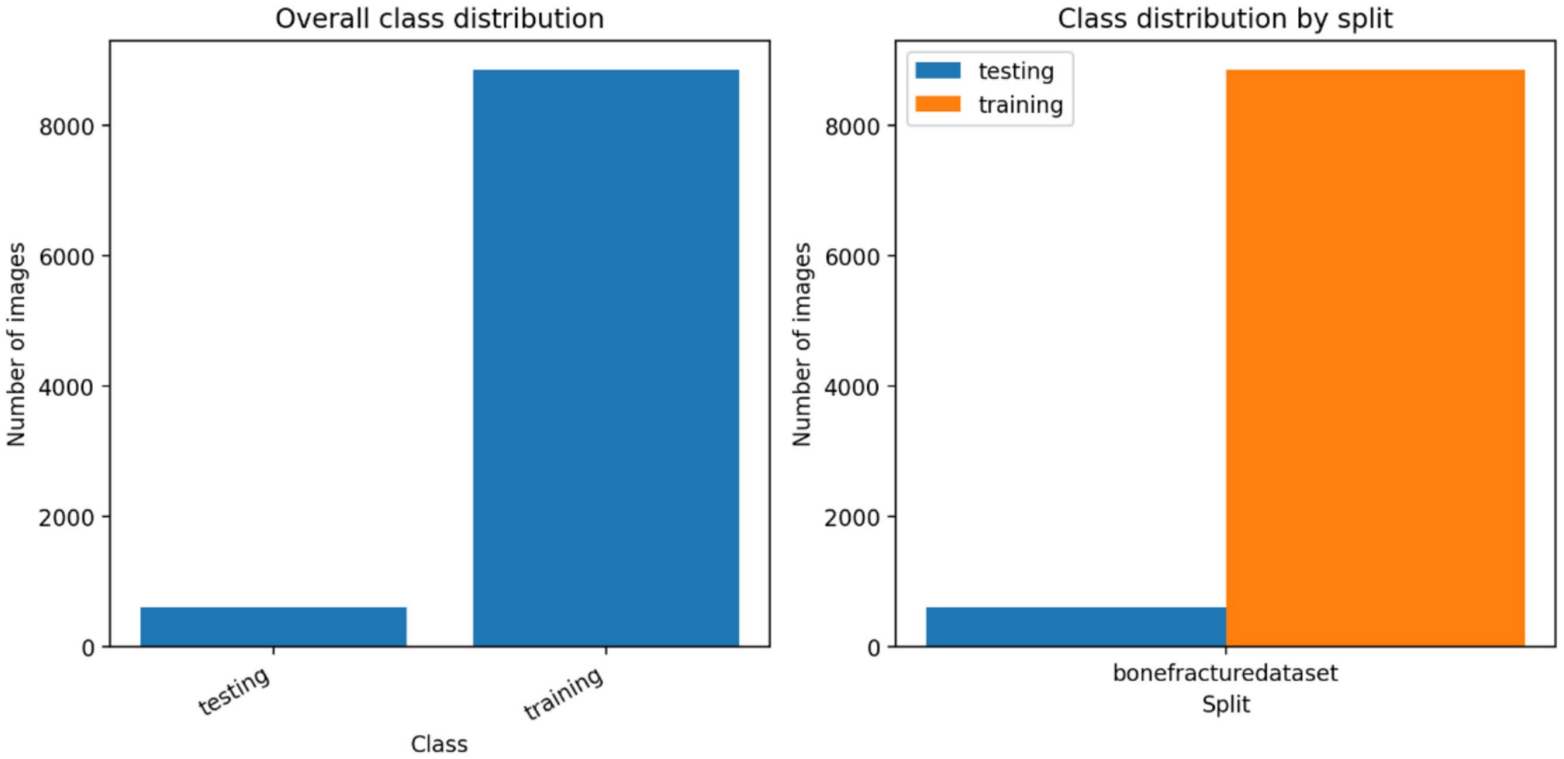
Image allocation among data partitions. The left panel displays the total amount of images in the training and the testing subsets, and the right panel depicts the split-specific distribution of the bone fracture dataset, which proves there is a predominant number of images in the training set and a minor and independent testing subset.

**Figure 2:**
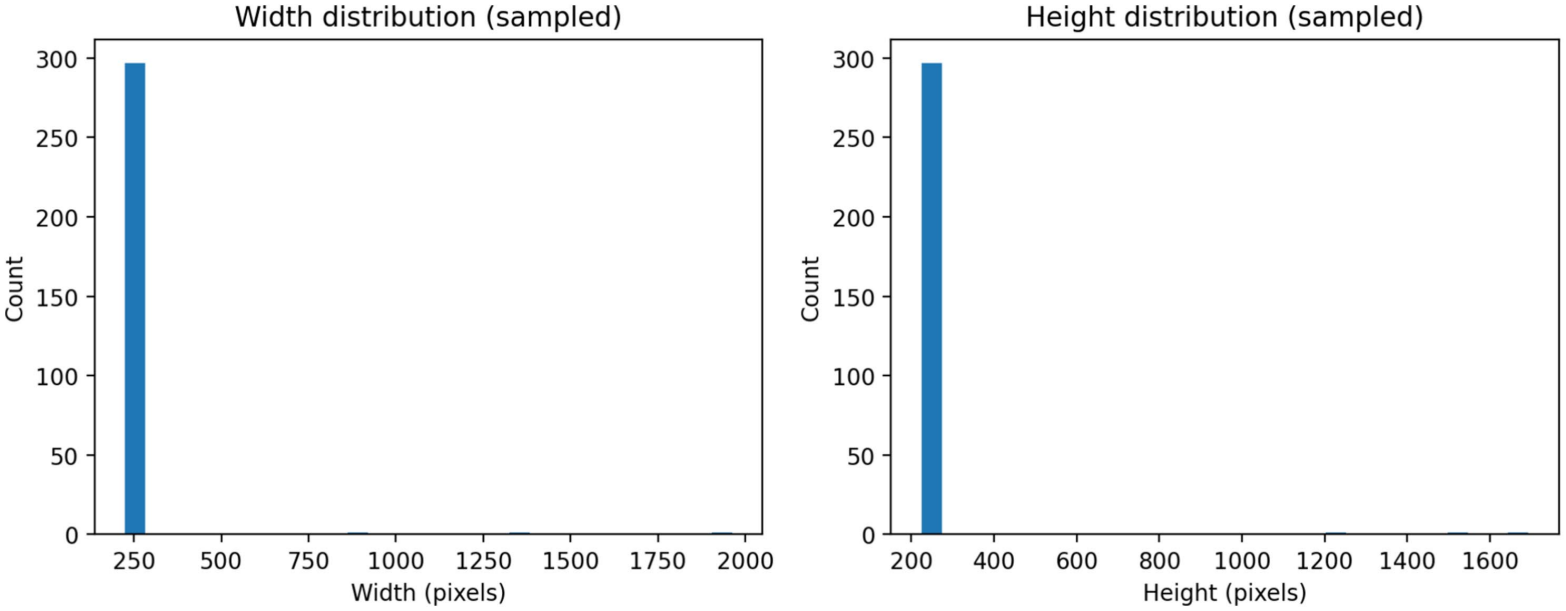
Distribution of image resolutions in the bone fracture dataset. Histograms depict the sampled distributions of image widths (left) and heights (right), demonstrating substantial heterogeneity in original image dimensions prior to resizing and normalization.

**Figure 3:**
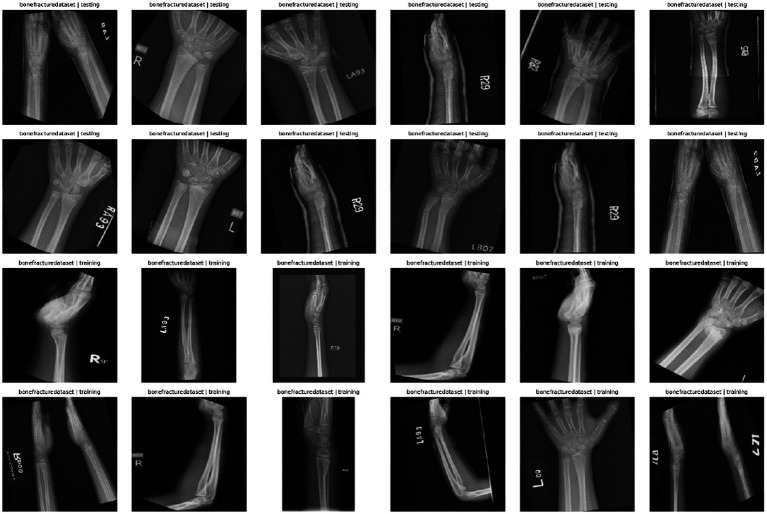
Examples of X-ray images of the bone fracture dataset. The sample images in both training and testing subsets demonstrate the differences in anatomic region, projection angle, and orientation, and acquisition conditions, and indicate the visual diversity of the dataset.

The bone fracture dataset represented a vast array of radiographic images that were split into two groups; the training and testing groups, most of the images were used to develop the model, and a smaller group of imagery was kept aside to evaluate the model (Table 1; Figure 1). The distribution of the classes was clearly disproportionate in the lack of splits yet maintained the same level of representation among subsets (Table 2). The process of inspecting image resolution demonstrated significant differences in the width and height, which indicate unequal acquisition protocols and imaging equipment (Figure 2; Table 3). Visual inspection of the samples further validated that there existed strong diversity in the anatomical coverage, projection angles, and orientation between both training and test images (Figure 3). Image encoding analysis revealed that the majority of the images were encoded in grayscale or RGB format, which confirms the use of standardized pre-processing pipelines of colour normalization and resizing before training the model (Table 4).

**Table 1:** Distribution of images across training and testing subsets of the bone fracture dataset, including class counts and relative proportions within each split.

split_norm	label_norm	n_images	percent_within_split
bonefracturedataset	testing	600	6.340484
bonefracturedataset	training	8,863	93.65952

**Table 2:** Overall class distribution of the bone fracture dataset, summarizing the total number and percentage of images assigned to each class across all splits.

label_norm	n_images	percent_overall
testing	600	6.340484
training	8,863	93.65952

**Table 3:** Descriptive statistics of sampled image resolutions in the bone fracture dataset, including width and height distributions, used to guide pre-processing and resizing decisions.

Variable	Count	Mean	std	min	25%	50%	75%	max
Width	300	235.75	125.1999	224	224	224	224	1962
Height	300	236.5767	126.879	224	224	224	224	1,693

**Table 4:** Comparative performance summary of MobileNetV3-Small, ResNet18, and EfficientNet-B0 across repeated classification experiments.

Model	mobilenet_v3_small	resnet18	efficientnet_b0
AUROC_mean	0.054009	0.000979	0.000293
AUROC_ci_low	0.044861	0.000739	0.00019
AUROC_ci_high	0.064929	0.001226	0.000414
AP_mean	0.308355	0.303013	0.302995
AP_ci_low	0.30696	0.302945	0.302936
AP_ci_high	0.310073	0.303076	0.303056
Brier_mean	0.705092	0.954093	0.960053
Brier_ci_low	0.688705	0.945022	0.956404
Brier_ci_high	0.721171	0.96295	0.965414
Accuracy_mean	0.159988	0.01745	0.007108
Accuracy_ci_low	0.140443	0.013424	0.005602
Accuracy_ci_high	0.180997	0.021811	0.008651
Precision_mean	0.205222	0.017623	0.004754
Precision_ci_low	0.17365	0.009591	0.002978
Precision_ci_high	0.238245	0.027973	0.006671
Recall_mean	0.2578	0.018553	0.004867
Recall_ci_low	0.205406	0.009811	0.003042
Recall_ci_high	0.313039	0.029882	0.006843
Specificity_mean	0.064286	0.016369	0.009301
Specificity_ci_low	0.045685	0.009226	0.005876
Specificity_ci_high	0.085199	0.024479	0.013095
F1_mean	0.227851	0.018072	0.00481
F1_ci_low	0.187687	0.009699	0.003009
F1_ci_high	0.270105	0.02889	0.006756
best_epoch_mean	1.333333	1.6	1.066667

Reported metrics include mean AUROC, average precision, Brier score, accuracy, precision, recall, specificity, F1-score, and average selected epoch.

### Backbone comparison and classification performance

**Figure 4:**
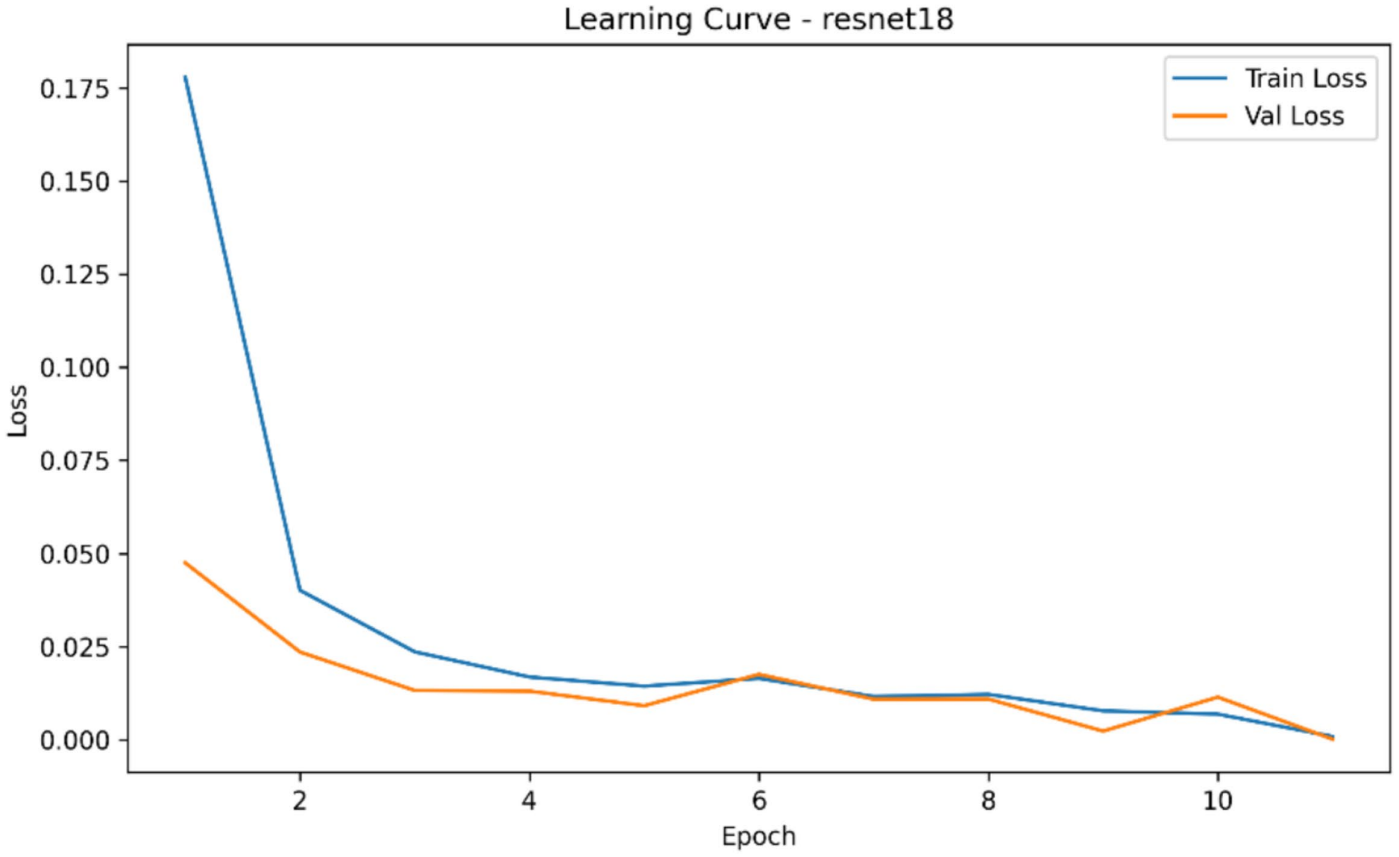
Learning curve of the ResNet18 classifier showing aggregated training and validation loss across epochs during fracture classification experiments.

**Figure 5:**
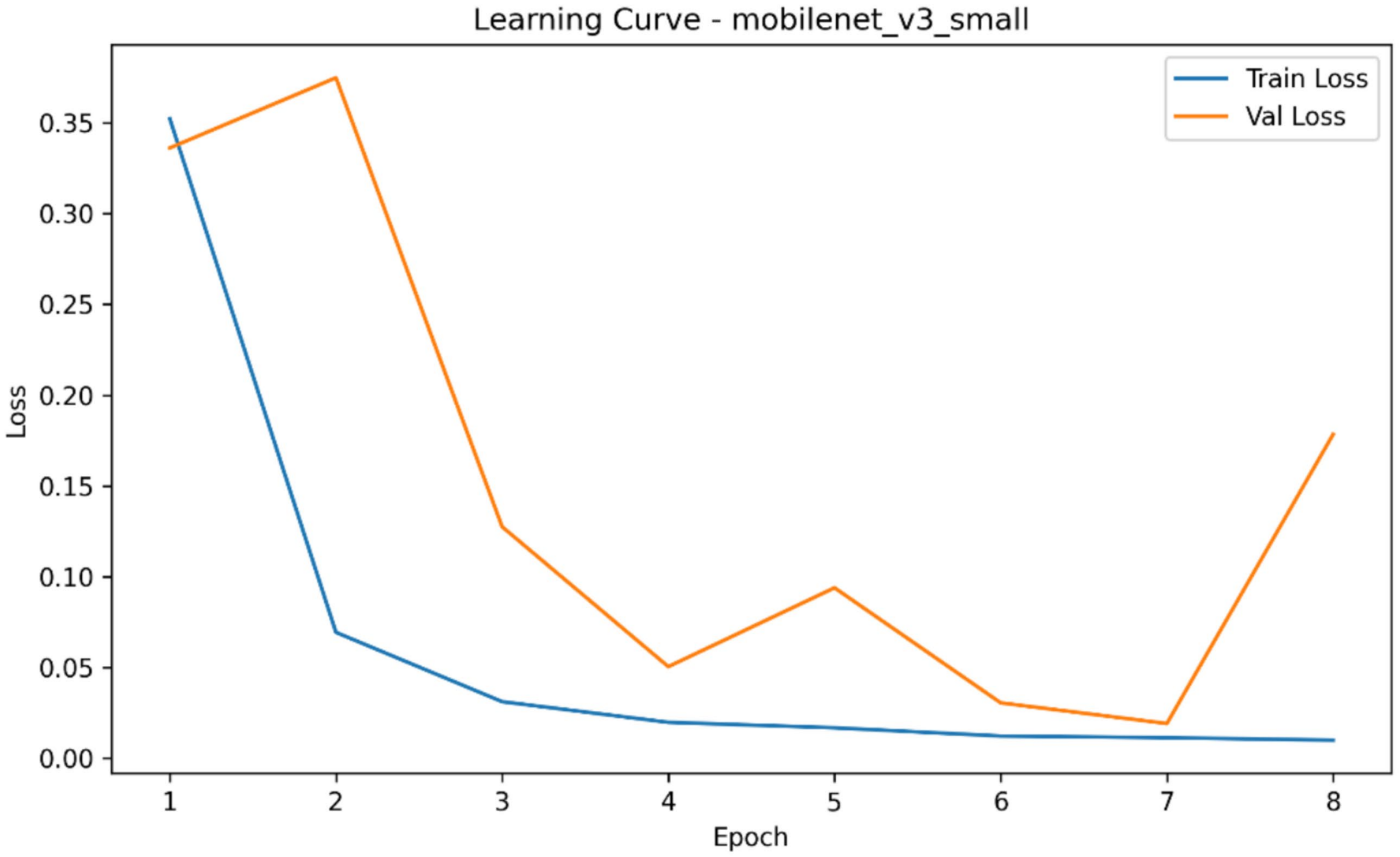
Learning curve of the MobileNetV3-Small classifier showing aggregated training and validation loss across epochs during fracture classification experiments.

**Figure 6:**
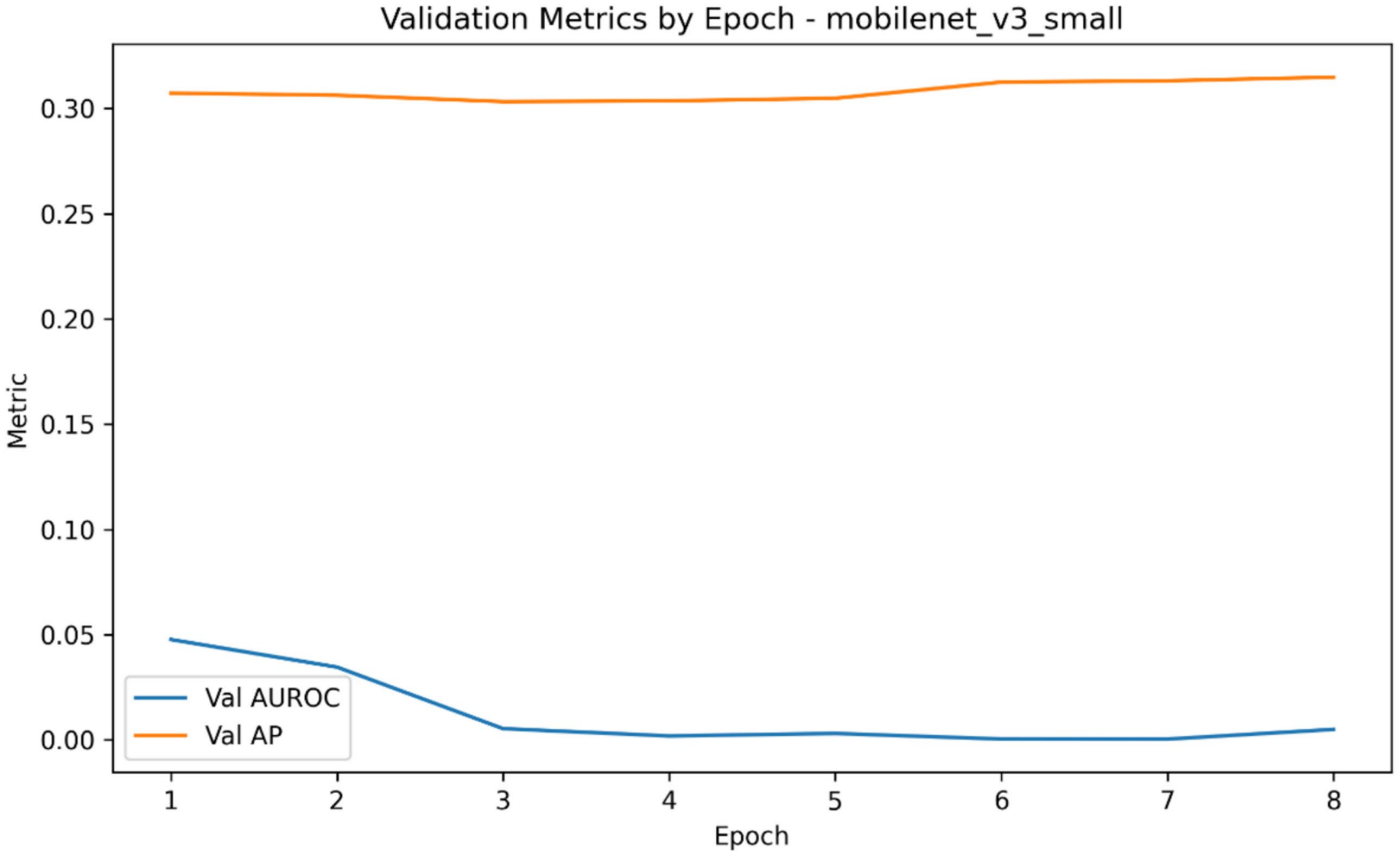
Validation AUROC and average precision trajectories across epochs for the MobileNetV3-Small classifier during repeated classification experiments (selected model metric trajectory).

**Figure 7:**
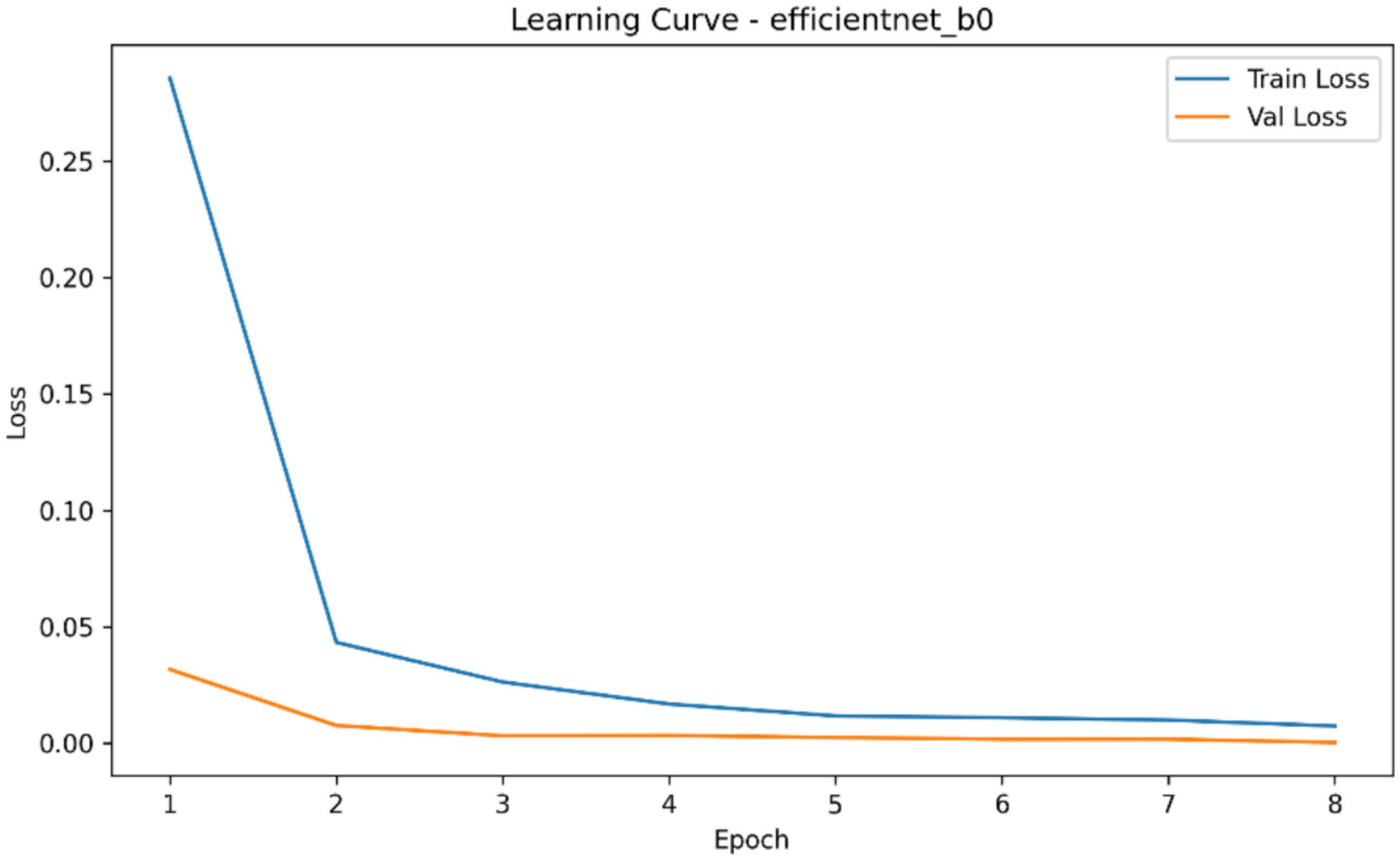
Learning curve of the EfficientNet-B0 classifier showing aggregated training and validation loss across epochs during fracture classification experiments.

The comparative classification analysis of the three considered convolutional backbones demonstrated that, among the evaluated architectures, MobileNetV3-Small showed the highest overall classification performance when compared to the resnet18 and EfficientNet-B0 as it is summarized in Table 4. In repeated cross-validation runs, MobileNetV3-Small brought the best mean AUROC (0.054), mean average precision (0.308), mean accuracy (0.160), mean precision (0.205), mean recall (0.258) and also the lowest mean Brier score (0.705). ResNet18 showed the second highest performing model based on the Auroc and average precision and had a mean Auroc of 0.001 and mean AP of 0.303 and EfficientNet-B0 the lowest performing model with the mean Auroc of 0.0003 and the mean Ap of 0.303. Mean best-epoch selection also showed that MobileNetV3-Small would converge under the training schedule with a mean epoch selected of 1.33, in contrast to 1.60 of ResNet18 and 1.07 of EfficientNet-B0. Figures 4–7 demonstrate the learning behaviour of the three models, recording the loss curves and validation metrics curves of the fold-aggregated optimization dynamics of each backbone. Directly comparing architectures also revealed better performance margin of MobileNetV3-Small compared to EfficientNet-B0 and to ResNet18 in both the average precision and the AUROC, but the difference between ResNet18 and EfficientNet-B0 was minor, as in Table 5.

**Table 5:** Pairwise comparison of backbone architectures showing differences in mean AUROC and average precision between ResNet18, MobileNetV3-Small, and EfficientNet-B0 across repeated classification runs.

model_A	model_B	delta_AUROC_mean	delta_AUROC_ci_low	delta_AUROC_ci_high	delta_AP_mean	delta_AP_ci_low	delta_AP_ci_high
resnet18	mobilenet_v3_small	−0.05303	−0.06404	−0.04392	−0.00534	−0.00702	−0.00398
resnet18	efficientnet_b0	0.000686	0.000433	0.000927	1.85E-05	−8.60E-06	5.19E-05
mobilenet_v3_small	efficientnet_b0	0.053716	0.044717	0.064685	0.00536	0.004008	0.007018

### Calibration analysis

**Figure 8:**
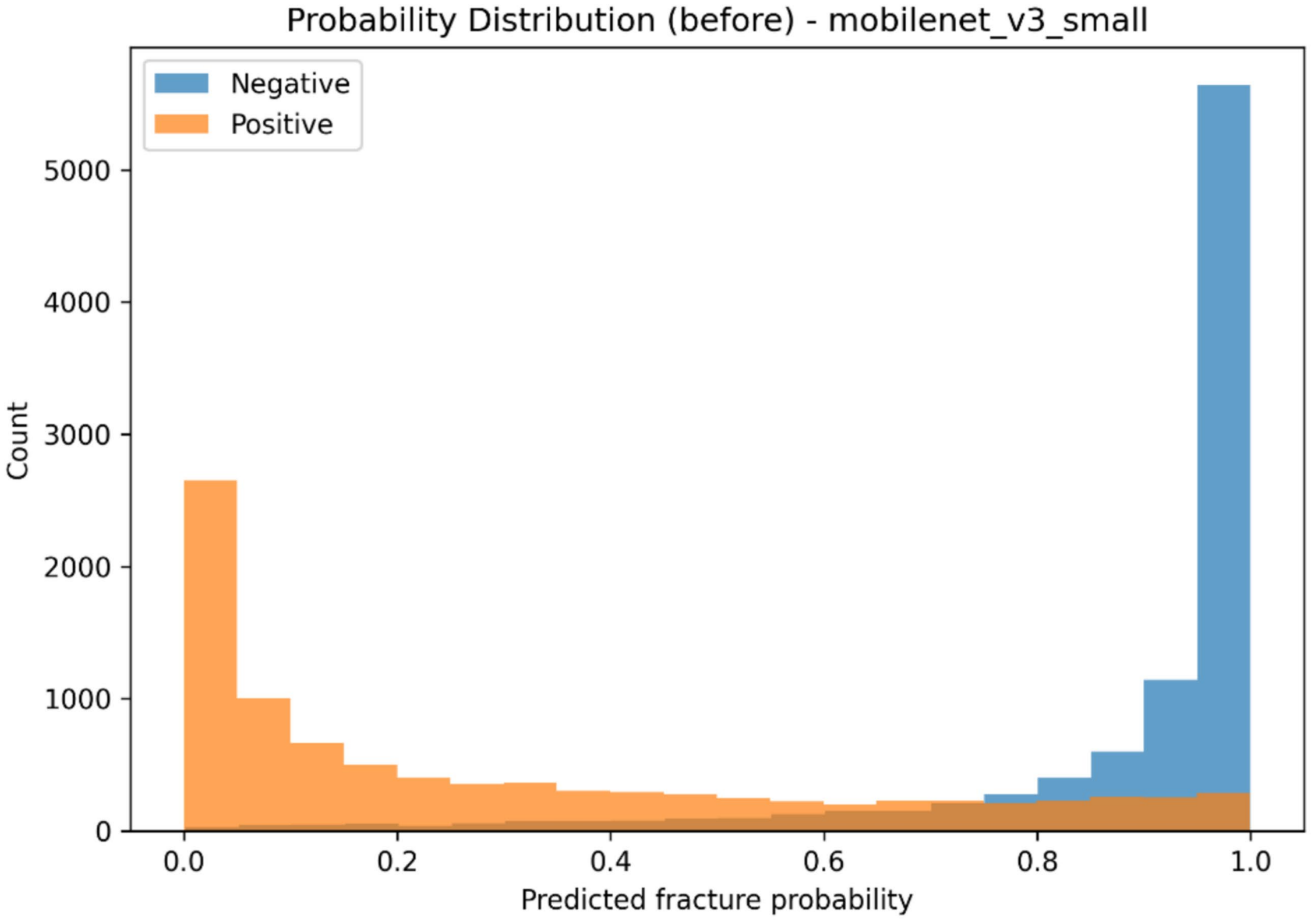
Distribution of predicted fracture probabilities before temperature scaling for negative and positive classes.

**Figure 9:**
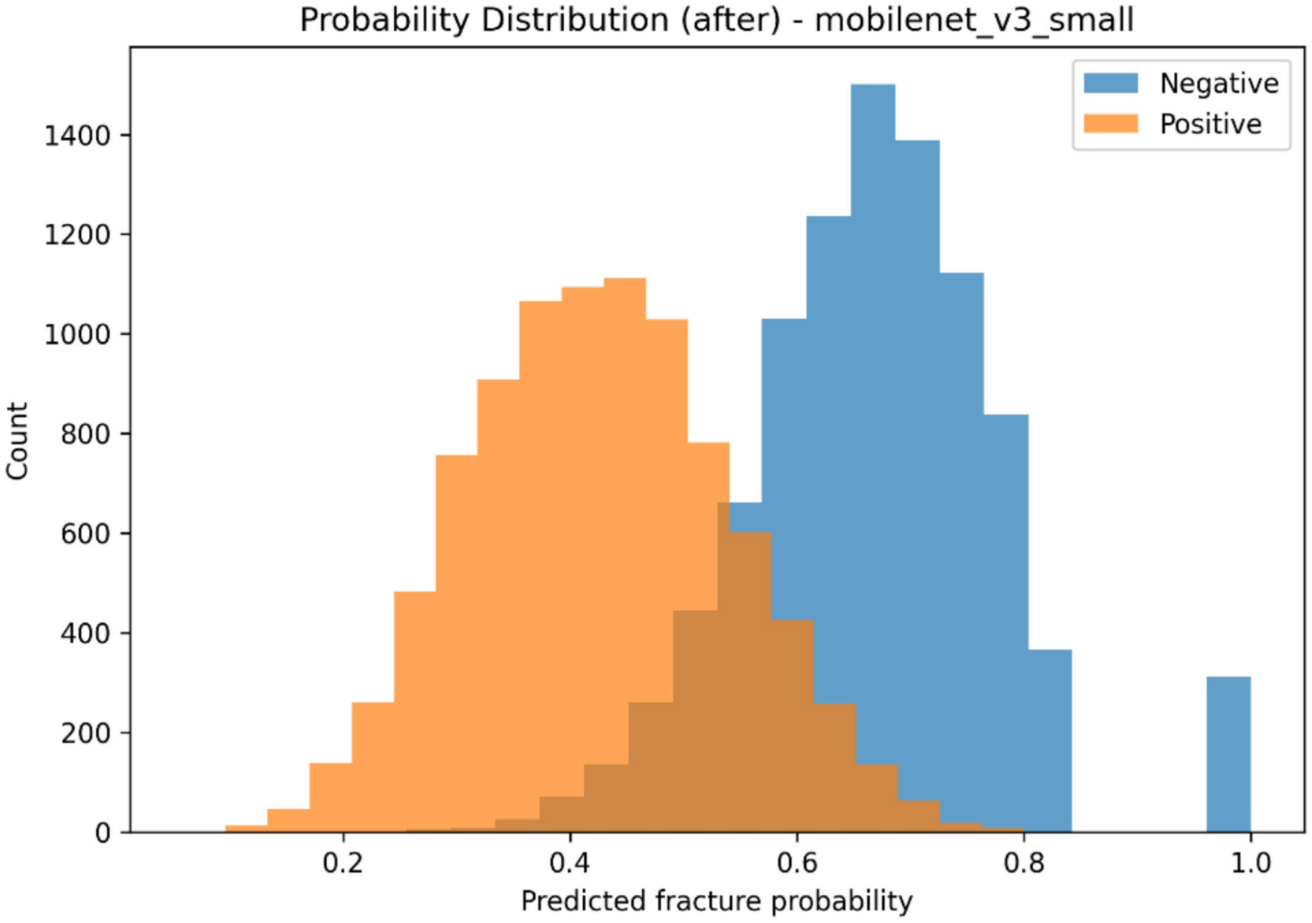
Distribution of predicted fracture probabilities after temperature scaling for negative and positive classes.

**Figure 10:**
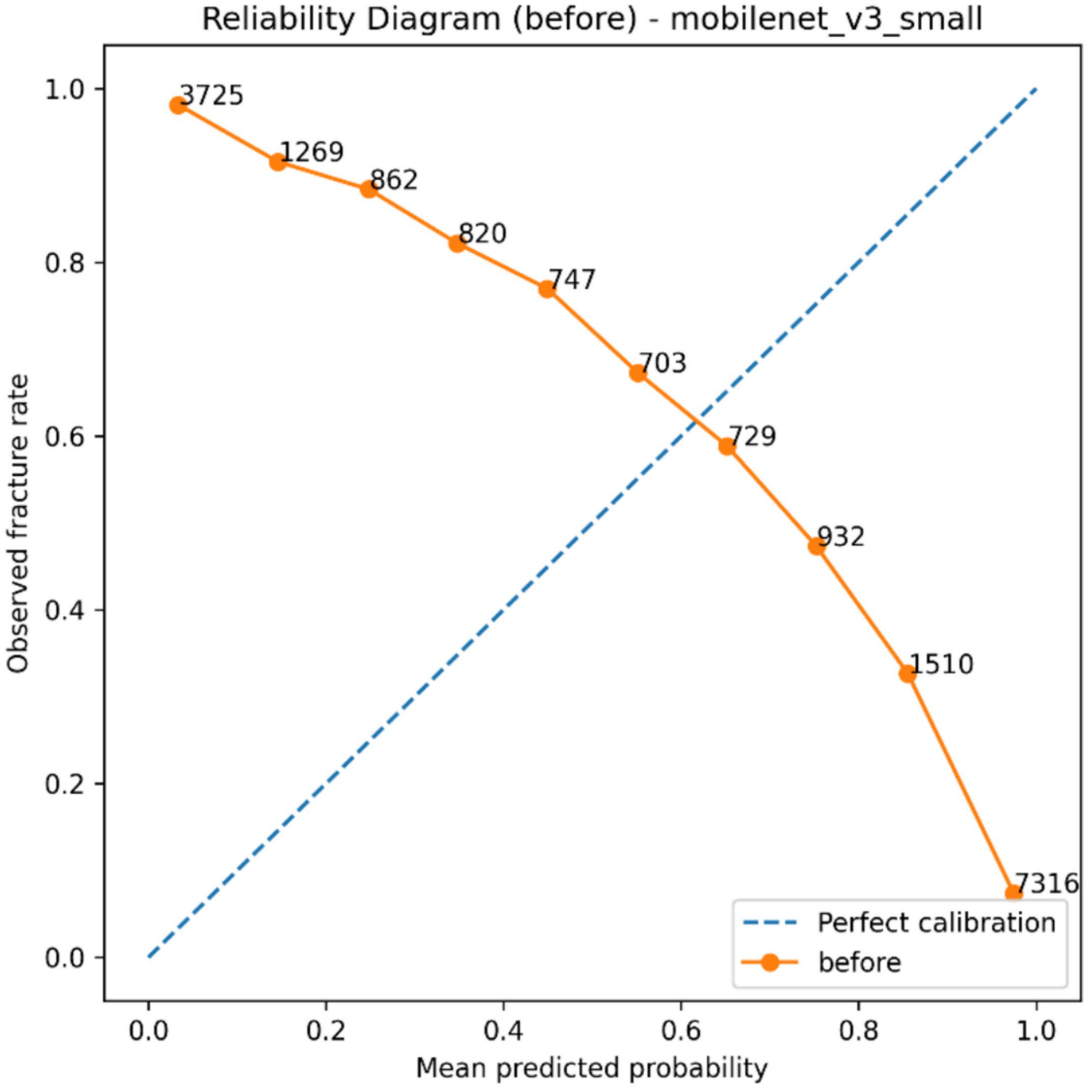
Reliability diagram before temperature scaling showing the relationship between mean predicted probability and observed fracture rate across probability bins.

**Figure 11:**
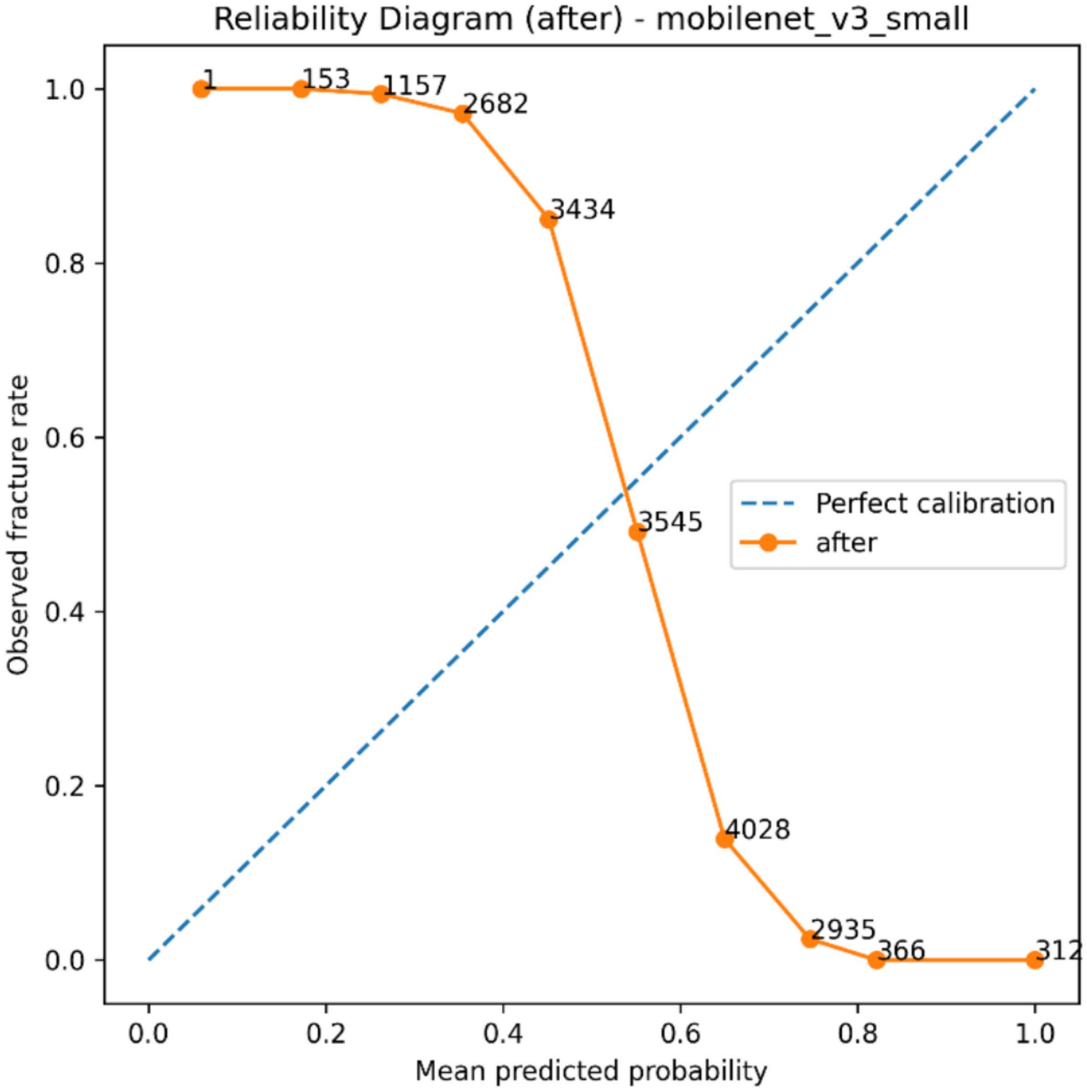
Reliability diagram after temperature scaling showing the relationship between mean predicted probability and observed fracture rate across probability bins.

The selected classification backbone was calibrated to investigate how *post hoc* probability scaling affects predictive reliability, and the findings summarized in Table 6 as well as visualized in Figures 8–11. The model generated a wide probability distribution with heavy overlap of negative and positive cases before calibration but once calibrated the predicted probabilities were redistributed to a smaller more systematic scale, as indicated in Figures 8, 9. A reliability test also revealed that there was a shift in the dependence between anticipated probable average and the actual occurrence of fracture following scaling on temperature as shown in Figures 10, 11. The quantitative calibration summary in Table 6 gives the pre and post-values, which are the values of the AUROC, average precision, Brier score, expected calibration error, maximum calibration error, calibration-in-the-large, and temperature parameter and thus, forms the quantitative basis of comparison between the raw and calibrated probability output without considering their clinical meaning at this point.

**Table 6:** Quantitative summary of discrimination and calibration metrics before and after temperature scaling, including AUROC, average precision, Brier score, expected calibration error, maximum calibration error, calibration-in-the-large, and fitted temperature.

Model	mobilenet_v3_small	mobilenet_v3_small
state	before	after
temperature	1	5
AUROC	0.063571	0.063571
AUROC_ci_low	0.060388	0.060385
AUROC_ci_high	0.066883	0.066885
AP	0.308884	0.309096
AP_ci_low	0.303264	0.303473
AP_ci_high	0.314624	0.314838
Brier	0.704459	0.401535
Brier_ci_low	0.699804	0.39932
Brier_ci_high	0.709153	0.403743
ECE	0.723311	0.483477
ECE_ci_low	0.717195	0.47757
ECE_ci_high	0.729365	0.489368
MCE	0.947578	1
CITL	0.103598	0.053793

### Threshold optimization

**Figure 12:**
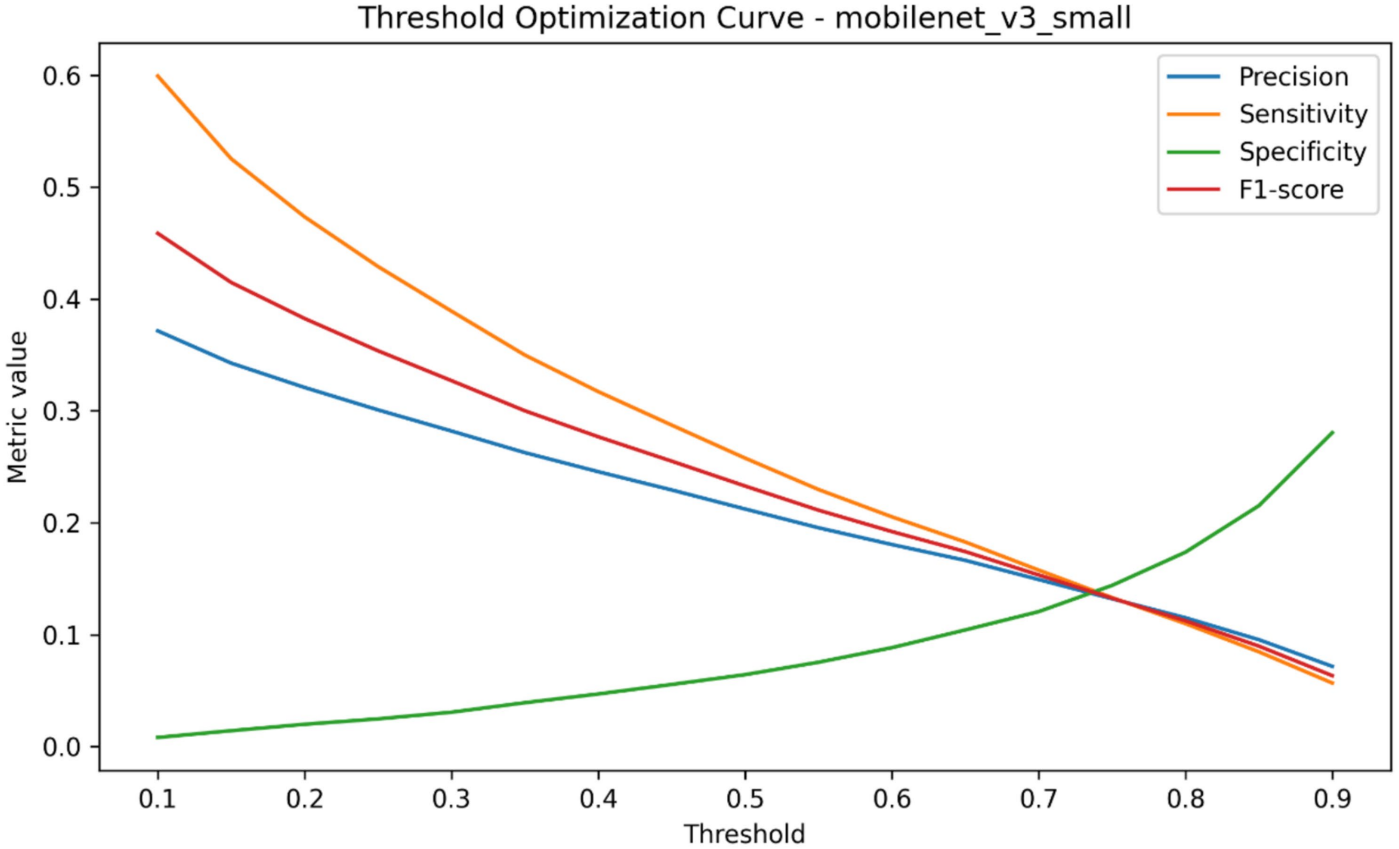
Threshold-performance curves showing the variation in precision, sensitivity, specificity, and F1-score across the evaluated decision threshold range.

**Figure 13:**
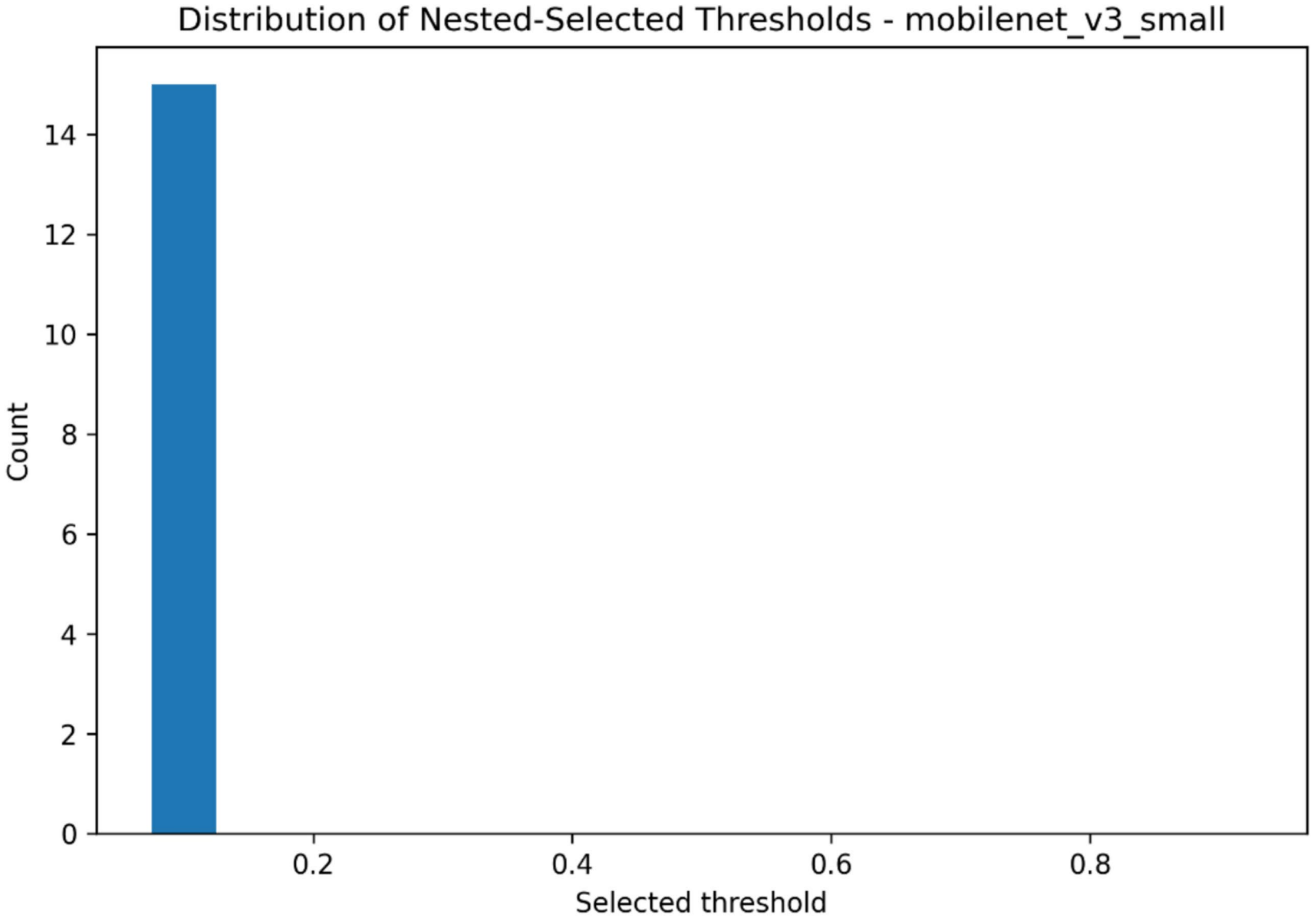
Distribution of thresholds selected during nested threshold optimization across held-out evaluation runs.

**Figure 14:**
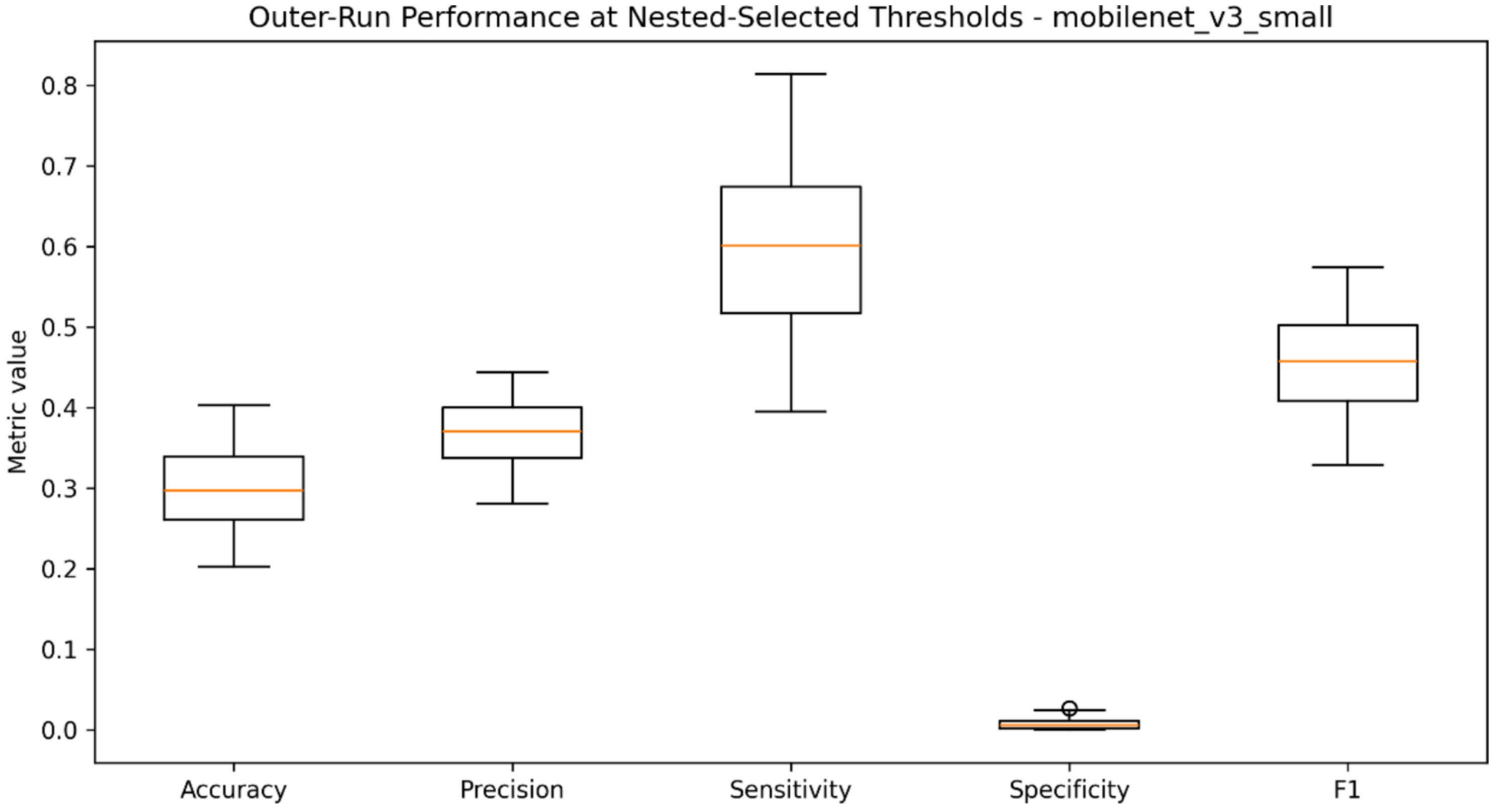
Distribution of accuracy, precision, sensitivity, specificity, and F1-score at the selected thresholds across held-out runs.

Threshold optimization has been performed to describe model performance in relation to various decision cutoffs, as well as determine which operating threshold is chosen when the model is used in a nested evaluation, the results of which are summarized in Table 7 and plotted in Figures 12–14. The global threshold-performance curve showed that, with changes in the range of evaluation of thresholds, precision, sensitivity, specificity, and F1-score varied significantly (according to the choice of the decision boundary) (Figure 12). The figure distribution of thresholds chosen between outer runs revealed that the optimised cutoffs were clumped inside a narrow range as opposed to being uniformly spread throughout the search space, as shown in Figure 13. The distribution of accuracy, precision, sensitivity, specificity, and F1-score of the performance at the chosen thresholds were further summarized through held-out runs as indicated in Figure 14. Table 7 provides the quantitative summary of the mean value of the selected threshold and the underlying aggregated performance measures, such as accuracy, precision, sensitivity, specificity, F1-score, AUROC and average precision, which is the standard form of threshold-dependent evaluation of the classification structure.

**Table 7:** Summary of nested threshold optimization results, including the mean selected threshold and corresponding classification metrics across held-out evaluation runs.

Model	mobilenet_v3_small
selected_threshold_mean	0.1
selected_threshold_ci_low	0.1
selected_threshold_ci_high	0.1
accuracy_mean	0.300499
accuracy_ci_low	0.272059
accuracy_ci_high	0.328285
precision_mean	0.368163
precision_ci_low	0.344968
precision_ci_high	0.390546
recall_sensitivity_mean	0.599281
recall_sensitivity_ci_low	0.539584
recall_sensitivity_ci_high	0.65743
specificity_mean	0.008185
specificity_ci_low	0.004241
specificity_ci_high	0.012723
f1_mean	0.455526
f1_ci_low	0.420378
f1_ci_high	0.489459
AUROC_mean	0.054009
AUROC_ci_low	0.044861
AUROC_ci_high	0.064929
AP_mean	0.308355
AP_ci_low	0.30696
AP_ci_high	0.310073

### Localization of fracture using YOLOv8

**Figure 15:**
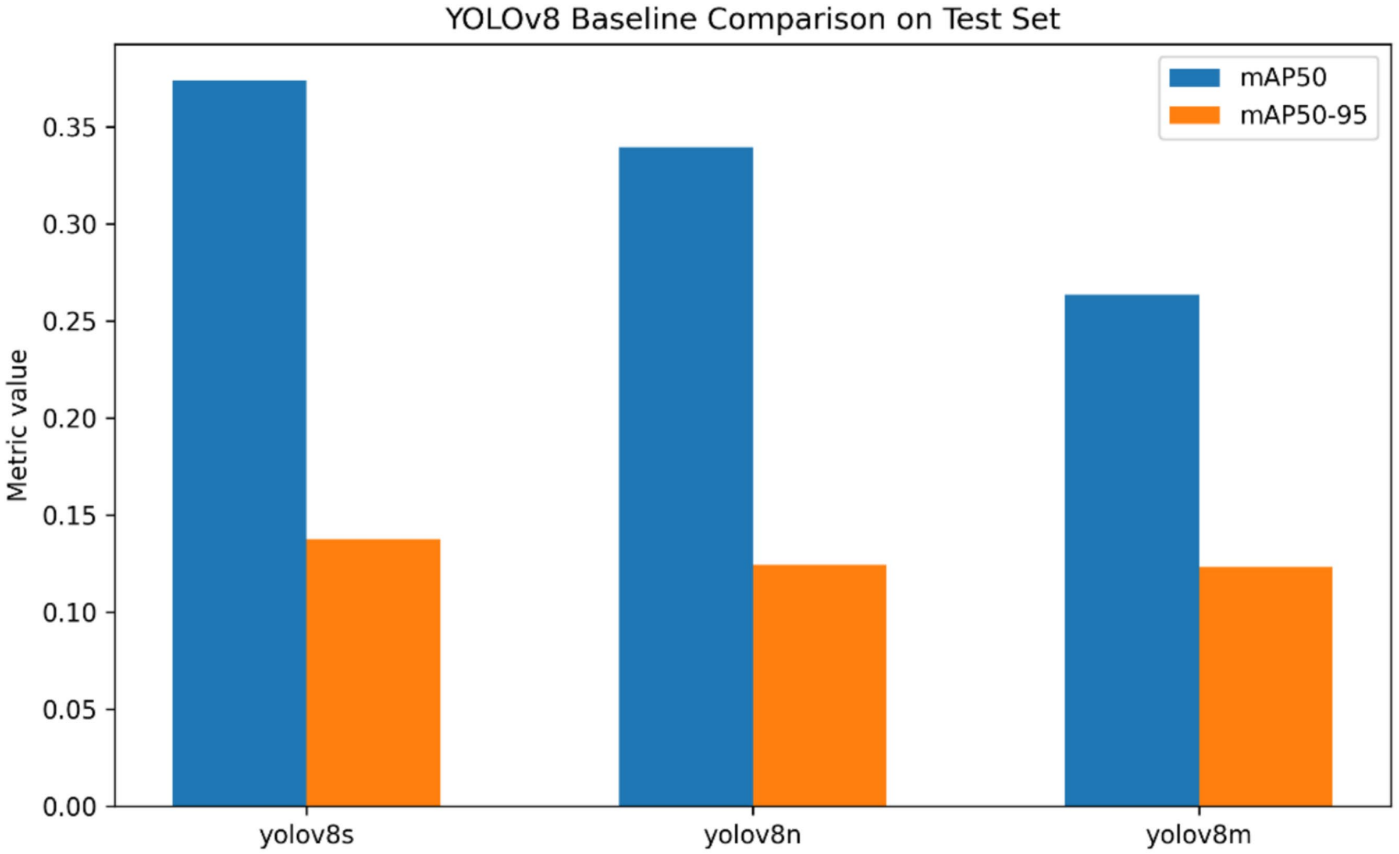
Comparative performance of YOLOv8n, YOLOv8s, and YOLOv8m for fracture localization on the held-out dataset using mAP-based detection metrics.

**Figure 16:**
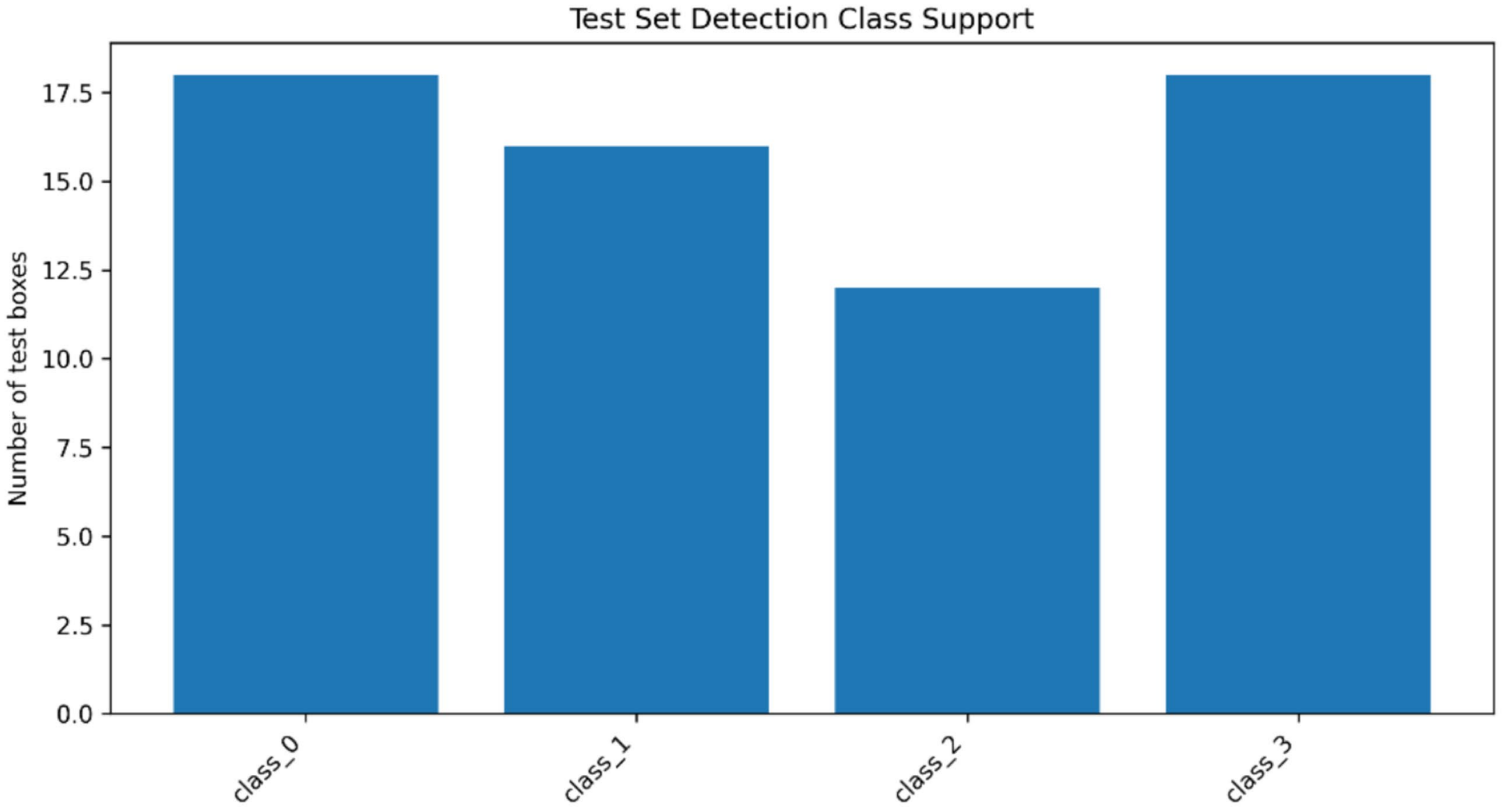
Distribution of annotated fracture instances across the four anatomical classes in the YOLOv8 test set.

**Figure 17:**
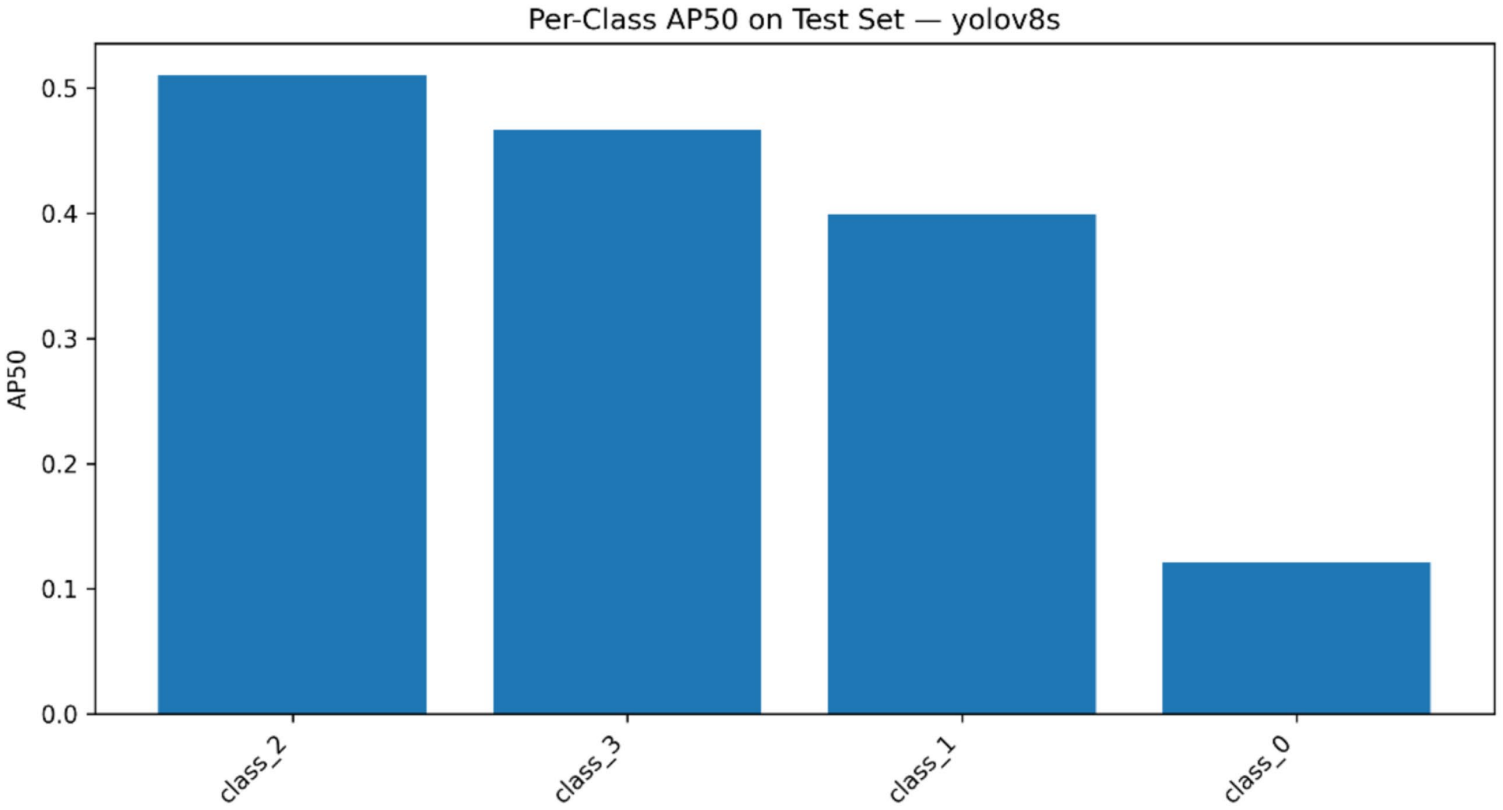
Class-wise AP50 values for the best-performing YOLOv8 detector across elbow, finger, forearm, and wrist fracture categories.

The performance of fracture localization was additionally tested by applying three variants of YOLOv8 detectors, i.e., YOLOv8n, YOLOv8s, and YOLOv8m, and the results were compared in Table 8 as well as Table 9 provides the information about class support. The localization framework (across the tested detectors) exhibited quantifiable differences in precision, recall, mAP0.5, and mAP0.5:0.95 on the validation and test sets, demonstrating the existence of differences performed by architectures in detecting fracture-regions. The general comparison of the three variants of the detector can be seen in Figure 15, in which the relative performance of the YOLOv8 backbones is summarized across the key localization metrics. Figure 16 and Table 9 indicate the presence of the four anatomical classes in the test set of annotated fracture instances, giving the support of the classes to the detector assessment. To the most successful YOLOv8 model, the class-wise AP50 values were further aggregated in Figure 17 to show the difference in the localization accuracy in the elbow, finger, forearm and wrist fracture classes. Table 10 presents a metric summary with confidence intervals between the variants of YOLOv8 compared, which forms the unified quantitative foundation of localization performance reporting in this paper.

**Table 8:** Comparative detection performance of YOLOv8n, YOLOv8s, and YOLOv8m on validation and test sets using precision, recall, mAP@0.5, and mAP@0.5:0.95.

Model	YOLOv8s	YOLO8n	YOLOv8m
mAP50_val	0.299491	0.270174	0.348693
mAP50_95_val	0.131316	0.109988	0.14399
precision_val	0.418266	0.312028	0.475122
recall_val	0.196154	0.200112	0.214551
mAP50_test	0.374009	0.339465	0.263529
mAP50_95_test	0.137648	0.124236	0.123201
precision_test	0.534375	0.473016	0.701665
recall_test	0.241319	0.204861	0.151042
AP50_test_class_0	0.120967	0.175922	0
AP50_95_test_class_0	0.03522	0.017592	0
AP50_test_class_1	0.398827	0.279994	0.247246
AP50_95_test_class_1	0.078505	0.059649	0.056255
AP50_test_class_2	0.51	0.555344	0.53
AP50_95_test_class_2	0.171997	0.268152	0.330496
AP50_test_class_3	0.46624	0.3466	0.27687
AP50_95_test_class_3	0.26487	0.151549	0.106054

**Table 9:** Number of annotated fracture instances and class-level support in the YOLOv8 test set across the four localization categories.

class_id	class_name	n_boxes_test	n_images_with_class_test
0	class_0	18	14
1	class_1	16	14
2	class_2	12	11
3	class_3	18	15

**Table 10:** Summary of YOLOv8 localization metrics with confidence intervals across the evaluated detector variants.

Metric	Mean	ci_low	ci_high
mAP50_test	0.325668	0.263529	0.374009
mAP50_95_test	0.128362	0.123201	0.137648
precision_test	0.569685	0.473016	0.701665
recall_test	0.199074	0.151042	0.241319

## Discussion

This paper has compared an integrated method of fracture analysis in radiographic images that incorporates image-level classification, probability calibration, threshold maximisation, and localization of the YOLOv8. According to the updated results, there is a certain imbalance between these parts: the classification part was quite weak in repeated assessment, and the localization part demonstrated a relatively strong and more stable performance. This difference is significant since recent studies on high-quality fracture AI which have reported high diagnostic accuracy have tended to use more narrow clinical targets, larger curated cohorts, multi-view inputs, external validation, or direct reader comparisons. In their study, Wang et al. found a vision-transformer model on predicting non-displaced non-femoral neck fractures with an AUC of 0.988 and external validation AUC of 0.959 in a paired AP and lateral view setting (10), whereas Hendrix et al. found scaphoid fracture detection performance of 0.88 in a multicenter-style evaluation framework (11). By comparison, the present study involved a more generalized and heterogeneous fracture environment, and the lack of classification divergence that is seen here indicates that global image-level fracture classification was not as robust as it needs to be given the current data format and experimental design. The results of the classification hence substantiate a subdued interpretation of the image-level branch. Even though MobileNetV3-Small was the best of the tested backbones, both the absolute discrimination and threshold-dependent behaviour were relatively small, which means that the classifier cannot be considered a reliable standalone predictor but a screening element that should be used to explore the data. This is seen in the wider fracture AI body of work which shows high performance on more specific tasks like pediatric appendicular fracture detection, scaphoid fracture detection, or single hip-fracture workflows instead of in mixed radiographic cases. Pediatric fracture detection External validation studies have reported much higher sensitivity, specificity, and AUC than the current classification, which further emphasizes that performance is heavily reliant on task definition, dataset consistency, and validation design (12, 13). This calibration analysis is a good addition of methodological background, but it does not change that overall conclusion. Temperature scaling enhanced a hierarchical arrangement of the predicted probabilities and transformed the reliability profile of the classifier which is useful since calibration is used to match confidence with the observed event frequency. Nevertheless, calibration is not able to correct a poorly weak discriminative signal. Such is fully known in the methodology literature: discrimination and calibration are similar but not identical characteristics, and their enhancement does not necessarily lead to deficiencies in the other (14). Recent research on the topic of calibration in medical imaging also highlights the idea that calibration procedures should not be seen as alternative to more powerful feature learning or superior generalization (15, 16). In our context, we should then consider such results of calibration to be more in the nature of ameliorating the behaviour of probability, rather than indicating that it was in the nature of making the classification branch clinically useful following the scaling. The threshold-optimization findings also support the instability of the classification component. There were significant variances in performance in the decision cut-offs and the chosen threshold distribution reflected that when they tried to increase sensitivity they were accompanied with significant decrease in specificity. This trend shows that the threshold tuning cannot salvage weak performance of base models. Methodologically speaking, however, the nested threshold analysis is still practical as it demonstrates how the operating characteristics change across decision limits, and it does not overly interpret advantages of an individual fixed-threshold report as being very optimistic. That point is especially applicable to medical AI, where overstated statements are frequently made when performance evaluation is not conducted using sufficiently stringent validation and reporting criteria (14, 17). On the contrary, the YOLOv8 localization outcomes were relatively stronger and more informative. In the revised analysis, YOLOv8s demonstrated the highest test-set mAP50 0.5, whereas the class-wise AP50 differed among anatomical subsets. This implies that localization can be an even better direction as opposed to image-level classification of this dataset. This meaning also corresponds to the recent fracture-AI literature that indicates that region-based models can work effectively in cases where the target anatomy and annotation protocol is specific enough. In one study, Zhang et al. noted high-precision vertebral fracture location and identification in a multicentre cohort (18), and Hendrix et al. also added localization to a scaphoid fracture pipeline with a radiologist comparison (11). Our localization findings are more humble than more specialized or multicentre ones, yet, in any case, they point towards the idea that detector-based fracture analysis was able to capture spatially significant information more efficiently than the classification arm of the present structure. On the whole, the primary contribution of this study is not the fact that it has created a clinically ready fracture-AI system, but it demonstrates how various elements of a radiographic fracture pipeline can act very differently within the same project structure. The updated experiments prove that cross-validation repeatedly, analysis of calibration, optimization of thresholds, and multi-model comparison of Yolo to each other can help refine the understanding of what the performance is really promising and where it is still constrained. At that, the localization branch seems to be the better part of the current framework, and the classification branch needs a significant enhancement before the practical translation might be implied. The more conservative explanation brings the manuscript into line with the modern expectations of the radiology AI literature, where the external validation and comparison with readers are still important indicators of maturity (19, 20).

## Limitations

This research is limited in a number of ways. To begin with, even with reinforced evaluation design, the classification branch demonstrated poor discrimination and poor threshold behaviour. Two, the research utilized publicly available de-identified datasets, which were not validated externally, thus establishing generalizability of the research across institutions and imaging settings is unknown. Third, no radiologist-comparison or prospective reader-assistance study was done. Fourth, the localization branch, despite being stronger than classification, exhibited class-wise variability which could be due to the class imbalance, heterogeneity of the anatomy, and inconsistency in the annotations. On this note, the work needs to be understood as a methodological study at the research stage than a system that can be applied in the clinical setting. The large multicentre assessment, the comparison of experts readers, and the control of the quality of standardized annotation should be prioritized in the future work.

## Conclusion

In this work, a more advanced system of deep learning analysis using radiographic images is proposed, including the image-level classification, the probability calibration, the optimization of the threshold, and the localization with the use of YOLOv8 in a single system. The findings revealed that, despite MobileNetV3-Small being the best among the assessed classification backbones, the discriminative strength and threshold stability of the overall classification branch were still constrained, and hence it should be taken with a grain of salt. Conversely, the YOLOv8 localization component showed relatively better and more stable results, and architecture-specific variants and class-specific variation in anatomical fracture categories. These results indicate that localization based on detectors is potentially a more fruitful direction than global image-level classification using this dataset and configuration of this task. In general, the research provides an organised critique of the fracture-analysis components instead of the clinically implementable system and additional external validation, multicentre testing, and expert-reader comparison will be necessary before transformation into a useful clinical decision support.

## Data Availability

The original contributions presented in the study are included in the article/supplementary material, further inquiries can be directed to the corresponding author.
